# The Role of Adverse Childhood Experience on Depression Symptom, Prevalence, and Severity among School Going Adolescents

**DOI:** 10.1155/2020/5951792

**Published:** 2020-03-18

**Authors:** Mekonnen Tsehay, Mogesie Necho, Werkua Mekonnen

**Affiliations:** ^1^Department of Psychiatry, College of Medicine and Health Science, Wollo University, Wollo, Ethiopia; ^2^Department of Psychiatry, College of Health Science, Mekelle University, Mekelle, Ethiopia

## Abstract

**Methods:**

A cross-sectional school-based study was employed. Five hundred forty-six secondary school students were selected using multistage sampling technique from 5 selected secondary schools. We obtained retrospective information on adverse childhood experiences of adolescents by ACEs, self-reported 10-item questionnaire, and current depression prevalence and severity by PHQ-9. Multivariate linear regression models were used to estimate child depression severity by retrospective ACE count.

**Results:**

Among the 546 adolescents who participated in this study, 285 (50.7%) of the participants answered yes to at least one or more questions among the total 10 questions of ACEs. Experiences of ACEs increased the risk for depressive symptoms, with unstandardized *β* = 1.123 (*β* = 1.123, 95% CI (0.872, 1.373). We found a strong, dose–response relationship between the ACE score and the probability of lifetime and recent depressive disorders (*p* < 0.0001).

**Conclusions:**

The number of ACEs has a graded relationship to both the prevalence and severity of depressive symptoms. These results suggest that exposure to ACEs is associated with an increased risk of depressive symptoms up to decades after their occurrence. Early recognition of childhood abuse and appropriate intervention may thus play an important role in the prevention of depressive disorders throughout the life span.

## 1. Background

Adverse childhood experience (ACE) is a term used to describe a wide range of stressful or traumatic events, including neglect, abuse, and household dysfunction such as growing up with family members who have substance use disorders, mental health problems, or intimate partner violence. Extreme economic adversity, bullying, school violence, and community violence are other commonly encountered ACEs [1, 2].

The US Federal Child Abuse Prevention and Treatment Act (CAPTA) defines child abuse and neglect as, at a minimum, any recent act or failure to act on the part of a parent or caretaker, which results in death, serious physical or emotional harm, sexual abuse, or exploitation, or as an act or failure to act which presents imminent risk of serious harm [3].

According to WHO report [4], child maltreatment is the abuse and neglect that occurs to children under 18 years of age. It includes all types of physical abuse, emotional abuse, sexual abuse, physical neglect, emotional neglect, household violence, household mental illness, the household member being incarcerated, household member substance use, and parental divorce/instability, which results in actual or potential harm to the child's health, survival, development, or dignity in the context of a relationship of responsibility, trust, or power. Exposure to intimate partner violence is also sometimes included as a form of child maltreatment [5].

Adverse childhood experiences (ACEs) have an incredible impact on future violence harassment and enactment and lifelong health [6]. ACEs are strongly associated with the development of a child and a wide range of health problems throughout the individual's lifespan [7].

When an individual is exposed to a stressful situation, the fight, flight, or freeze response floods the brain with corticotrophin-releasing hormone (CRH). This response is normal and protective in stressful situations [2]. However, if a child is continually exposed to ACEs, the brain also continually produces CRH, and this process results in the child being in a permanently heightened state of alertness, unable to return to the recovered state. Therefore, the child or adolescent is always at an increased level of stress [8, 9]. In this heightened neurological state, the adolescent is unable to think rationally and it is physiologically hard or impossible to learn.

Most recent kinds of literatures also showed that stress is most often associated with aversive states, which induces the release of hormones and neuropeptides including dynorphin, which activates kappa opioid receptors (KORs) in the central and peripheral nervous systems. Prolonged KOR signaling in response to chronic or uncontrollable stress intern can lead to the persistent expression of behavioral signs that are characteristic of human depressive disorders [10–12].

Extreme stress can disrupt the development of the nervous and immune systems. Children who have faced abuse and neglect are at increased risk in adulthood for depression, alcoholism, drug abuse, high-risk sexual behavior, chronic diseases, and even suicide [13]. As research shows that chronic stress plays a critical role in the development of hippocampal and medial prefrontal cortex deficits, which is the well-documented neural abnormalities in major depressive disorders [14].

Adverse childhood experiences (ACEs) include abuse, neglect, and well-established downstream health before the age of 18, and ACEs that have household dysfunction experiencing behavioral health problems in higher ACE scores help us to predict consequences over the life course. Adverse childhood experiences (ACEs) include a broad range of events that constitute childhood trauma [15].

They include verbal, physical, or sexual abuse as well as neglect or dysfunctional family conditions or events such as mental illness, substance abuse, domestic violence, or incarceration. ACEs have been linked to adverse health outcomes in adulthood, including depression, substance abuse, cardiovascular disease, and premature mortality [16].

Relationship between ACEs and increased risk of negative physical and behavioral health outcomes among adults in high-income countries [17] includes an increased risk of tobacco, alcohol, and drug abuse [18].

Social support may have a protective or buffering effect against the consequences of a stressful event by enhancing cognitive and emotional processing of the experience [19].

Improved knowledge on the relations between distinct types of ACEs and adolescent depression would afford insight into the development of psychological problems among young adolescents and the tailoring of mental health care to meet their needs. To our knowledge, ACES have been well studied worldwide, but this is the first study among the vulnerable population in Ethiopia.

The present study aims to retrospectively describe the prevalence of distinct types of ACEs (i.e., abuse, neglect, and household dysfunction among adolescents) and to examine whether these ACEs are differentially associated with the prevalence and severity of depression adolescence.

## 2. Methods and Materials

### 2.1. Study Design and Study Setting

The school-based cross-sectional study design was employed from April 2, 2018–May 30, 2018 G.C. This study was conducted with the Jimma town government and private secondary school students. Jimma is located at 357 km to the southwest of Addis Ababa. It is found in the Oromia Region of Ethiopia. Projecting from the 2007 census, Jimma town has a total population of 159,009, of whom 80,897 were males and 78,112 were females.

The town has six public and eight private high schools, and 9383 students had registered for grades 9, 10, 11, and 12 in the academic year of 2017/2018. Of these, 7292 were from public and 2091 were from private schools.

### 2.2. Study Population

All adolescent students aged 10-19 who were studying in selected Jimma town secondary schools and students who are willing and allowed by the parents to participate in the study were recruited. Adolescents who are found to have severe illness during the study and difficulties in communication to the data collectors were excluded.

### 2.3. Sampling Procedures

The sample was calculated by taking the prevalence of depression among high school students of Uganda which was 21.0% (7). We assume any particular outcome to be within a 5% margin of error and a 95% confidence interval of certainty (alpha = 0.05) and a 10% nonresponse rate. Based on these assumptions, the sample size of the study was calculated using a single proportion formula, and the final sample size was 546.

A multistage sampling technique was used. In the first stage, schools were stratified into two. All the government schools (*n* = 6) and private schools (*n* = 8) were numbered separately, and two random numbers were chosen from governmental (Jiren and Jimma preparatory) schools and three from private (Eldana, Sos and Tesfa Tewahdo) secondary schools. In the second stage, students of the selected governmental and private schools were stratified into grade levels from 9th to 12th separately.

**Figure 1:**
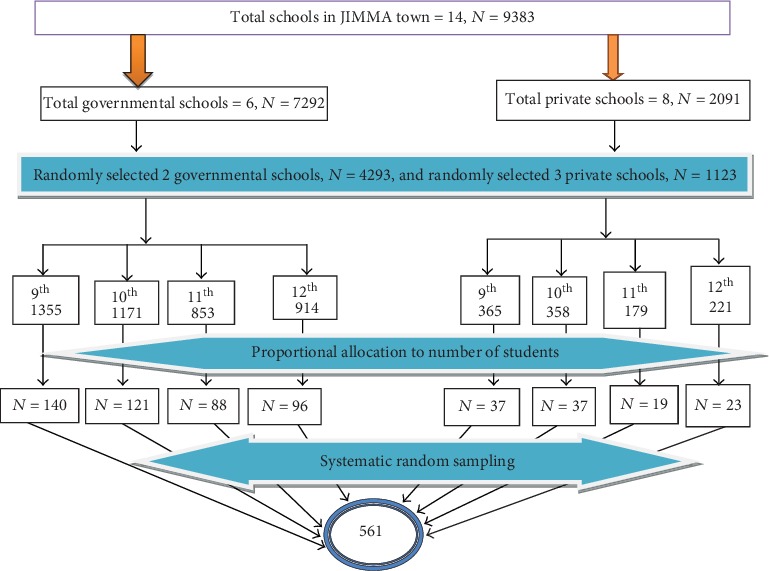
Schematic representation of study sample.

Finally, the sample size was allocated as per the number of students in each grade level using the proportional allocation method. After this, lists of students from 9th to 12th grade were obtained and the students were numbered consecutively in each class as per their roll numbers. This number was equally divided based on the number of sections in each class in a particular school. The sampling scheme from government and private schools is shown in Figure 1.

One day before data collection, selected adolescent students in the selected school were provided with information sheet about nature, purposes, benefits, and adverse effects of the study and consent form paper to take to their parents at home who then signed the consent forms if they allowed their child to participate in the study after they read information sheet. Students who are allowed to participate were given written informed assent and signed for their participation.

### 2.4. Predictors

Adverse childhood experiences exposure to ACE was assessed using the ACE questionnaire (3), which addresses 10-item ACEs under three categories: (1) abuse (emotional, physical, and sexual), (2) neglect (emotional and physical), and (3) household dysfunction (parental separation/divorce, violence against mother, household substance abuse, household mental illness, and incarceration of the household member). The ACE questionnaire is a reliable and valid measure of childhood adversity that has been used extensively in large-scale ACE studies. All questions about ACEs pertained to the respondents' first 18 years of life and were binary (yes vs. no). From these, variables were created to reflect any exposure to each ACE subtype (abuse, neglect, and household dysfunction). We also calculated a total ACE score for each participant (+1 for each of the 10 types of ACE reported). The ACE score was treated as a categorical variable (0, 1-3, or >3) to capture any potential nonlinearity in the relationship with depressive symptoms. This method of ACE score categorization has been used previously [20, 21].

### 2.5. Outcomes

#### 2.5.1. Depression Prevalence and Severity

Patient health questionnaire-9 for adolescents (PHQ-9A) screening tool has nine items and was validated in the Ethiopia adult population with a sensitivity of 86% and specificity of 67% (1). In other countries, PHQ-9 modified for adolescents was used and they validate (University of Washington) for this specific population. PHQ − 9 score > 10 had a sensitivity of 89.5% and specificity of 77.5% for detecting youth meeting DSM-V criteria for major depression (2).

#### 2.5.2. Covariates

In addition to sociodemographic variables, each student also reported to self-rated health questions, on a Likert scale with five points (“poor,” “fair,” “good,” “very good,” or “excellent”). Substance use questions were also incorporated to assess any abuse history and the Oslo 3-Item Social Support Scale was used to assess social support. A sum index was made by summarizing the raw scores, the sum ranging from 3 to 14. The questionnaires were prepared in English then translated into Afaan Oromo and Amharic languages for better understanding. Ten second-year MSC psychiatry students were assigned to supervise and disseminate the questionnaires. Before the data collection, one-day training had been given for the supervisors. The supervisors for completeness and consistency of responses had checked the filled questionnaires daily. Before the actual data collection, a pretest was made on 5% of the sample size in Agaro secondary school which is found in other zones near the study site, and necessary correction was taken after the pretest was done on the questionnaires. The reliability and validity of the instruments in our research were found to be very good, using a cut-off of 10 on the PHQ − 9A Cronbach′s alpha = 0.836.

#### 2.5.3. Data Processing and Analysis

Data were entered into Epi data 3.1 and exported to SPSS version 23.0. Before the data analyses were conducted, assumptions like normal distribution of continuous dependent variables, linear relationship with independent variables, test for independence, and multicollinearity, outlier, homoscedasticity, and normal distribution of residuals were verified using the histogram with the fitted normal curve, P-P-plot, scatter plots, Durbin-Watsons test, tolerance, variance inflation factor (VIF), and K-S tests. Descriptive analyses were computed.

Chi-square tests were performed to examine any differences in ACE prevalence (cumulative and individual ACEs) by gender and by depression symptoms, and multiple linear regression analysis was performed by using forward methods to examine the associations between independent variables and depressive symptoms. Statistical significance was defined at *P* < 0.05 (two-tailed).

## 3. Result

### 3.1. Description of Sociodemographics

Among the total number of 561 distributed questionnaires, 546 were filled completely and consistently making a response rate of 97.3%. The remaining 15 questionnaires were not included in the study as a result of nonresponse rates collected from all the sampled schools. Of these, 442 (81%) were studying in public schools and 104 (19%) in private schools. Adolescent females outnumbered the males. The majority of the participants were from the urban area. Their ages ranged from 14 to 19 years with a mean age of 16.83 years (SD ± 1.26). The majority of respondents, 211 (38.6%), were under age 16, 153 (28%) were age 17, and 182 (33.3) were above age 18. The majority (44%) were Muslims and about (38.8%) were Christians. Occupation of respondents' fathers was classified into four categories where 40 (7.3%) were laborers, 219 (40.1%) were merchants, 163 (29.9%) were government employees, and 124 (22.7%) were private employees. Likewise, respondents' mother occupation was under the same category with an additional category namely being a housewife, which accounted for 135 (24.7%) mothers out of the total number of mothers. Five classifications were used to categorize respondents' father and mother educational levels. Accordingly, 150 (27.5%) of the response for fathers' educational level were under the certificate and above category while the number of mothers' educational level for the same category was 102 (18.7%) (Table 1).

### 3.2. Adverse Childhood Experience among Adolescents

Among the 546 adolescents who participated in this study, 285 (50.7%) of the participants answered yes to at least one or more questions among the total 10 questions of ACEs. According to the three category of adverse childhood experience (ACE) from 332 female adolescents, 60 (18.1%) had been abused either physically or sexually, 58 (17.6%) had been neglected and 100 (30.4%) had been household dysfunction. And also from 214 male adolescents, 75 (34.6%) had been abused either physically or sexually, 43 (19.6%) had been neglected, and 73 (33.6%) had household dysfunction (Table 2).

### 3.3. Description of Social Support

Measurement by the Oslo 3-Item Social Support Scale revealed that 173 (31.7%) of respondents received poor social support, 211 (38.6%) of them had moderate social support, while 162 (29.7%) were have strong social support.

### 3.4. Description of Substance Use among Adolescents

This study shows that a significant number of students use alcohol (16.1%), followed by khat-chewing behavior (8.1%), and small number of student's use cigarette and other illicit drugs (shisha) (0.9%) each.

### 3.5. Description of Depressive Symptoms among Adolescents

**Figure 2:**
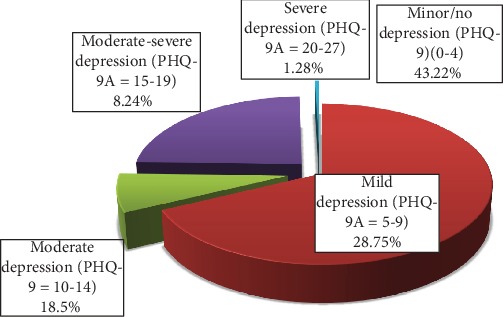
Prevalence and severity of depression among adolescents.

In this study, the prevalence of major depressive disorder was found to be 28% (with CI 24.5, 32.1) and that of other depressive disorders was 28.75%. Forty-three percent (43.22%) of children had no depression. Based on the severity scale, 28.8% had mild depression, 18.50% had moderate depression, and 8.24% had moderately severe depression. And 1.3% had severe depression. The prevalence of suicidal thought is 6.4% (with CI 4.4, 8.8), and the history of suicidal attempt is 7% (with CI 4.9, 9). And the prevalence of dysthymic symptoms is 55 (10.1%; with CI 7.5, 12.8) (Figure 2).

### 3.6. Effect of ACEs on Prevalence and Severity of Depression Symptoms

The prevalence of participants with a PHQ-9 score indicative of major depression was significantly higher among participants who reported for more than one ACE (ACEs 1 − 3 = 29.9%, ACEs > 3 = 62.3%) compared with participants who did not (19.7%). As shown in Table 3, dose-dependently, a number of participant with depression symptoms increase with ACEs count.

In the final linear model among many variables included in the multiple linear regression analysis, only gender, social support, residence, grade level, negative life events (ACE) score, and self-rated health were significantly associated.

Therefore, as shown in Table 4, from sociodemographic factors, being male decreases depressive symptoms by 1.107 units (*β* = −1.107, 95% CI (−1.906, −0.308) as compared to females, and coming from rural woredas of the Jimma zone increases PHQ-9A score by 1.174 (*β* = 1.174, 95% CI (0.137-2.211). Moreover, being in grade 9 and 10 decreases PHQ-9 score (*β* = −1.107 95% CI (−2.797, −0.960), and having experience a negative life event during childhood increases PHQ-9A score by 1.123 (*β* = 1.123, 95% CI (0.872, 1.373).

A unit change in Oslo-3 Social Support Scale score also decreases PHQ-9A score (depressive symptoms) by −0.273 (*β* = −0.273, 95% CI (−0.378, −0.168).

## 4. Discussion

To our knowledge, this is the first study examining the association between ACEs and depression in a vulnerable sample in Ethiopia. In this study, ACEs were highly prevalent (50.7%) and it was found higher in females than in males 52.1% and 48.6, respectively. Linear regression analyses also showed that it is a dose–response relationship between ACEs. More than 41% reported one up to three ACEs and 9.3% reported more than three ACEs. Our study supports the findings from the previous studies examining the ACE items in youth [22]. The current study shows lower prevalence of ACEs than the previous studies [7, 23, 24]; the slight difference may account for the population difference and questionnaire used, in which we used the 10 items, but some others use the 8- or 9-item ACE questionnaire. And also, we used yes/no for each of the 10 items, but Robert et al.'s study used the Likert scale having 5 response rates for each items [25].

There were a few significant differences in ACE prevalence by gender or grade level, which is consistent with previous research [23]. A worldwide meta-analysis, which was conducted across continents in 2012 at the Netherlands, which shows that there are no gender differences and a childhood abuse is a universal phenomenon, reported that the self-reported prevalence of childhood emotional abuse was estimated at 36.3% whereas the prevalence based on informant studies was 0.3%, or 3 per 1000 children [26].

The findings also demonstrate that adverse experiences of childhood are associated with prevalence and severity of depressive symptoms, which shows that by making other factors, gender, social support, residence, grade level, and self-rated health constant, have adverse experience negative life event during childhood increases PHQ-9A score by 1.123 (*β* = 1.123, 95% CI 0.872, 1.373). Adverse childhood experience plays a role as a prevalence and severity predictor of depressive symptoms. As the number of ACEs of a person has increased, so does the risk for depression symptoms. The graded association that emerged between number of ACEs and depressive symptoms is consistent with prior studies [25, 27–30]. A study done in Minnesota suggests that ACEs predicted the worsening of mental health. And another study done in Ireland shows ACE exposures (overall, subtype, or ACE scores) were associated with higher odds of depressive symptoms [21, 31].

Those with higher levels of ACEs had greater levels of stress and lower levels of social support [32]. Early adverse childhood experiences may lead to early childhood mental health, chronic medical, and social development problems [33].

Study limitations include the following. It is difficult to establish causality between ACEs and depression, especially among adults, given that their responses may be affected by recall or social desirability biases.

## 5. Conclusion

Our study findings have potential implications for future research and practice. Our analysis expands the existing body of evidence addressing the association between ACEs and adolescents' prevalence and severity of depression. Health systems should support the routine use of ACEs as a mental health screening tool; this should be accompanied by training clinicians to implement the ACE scale during clinical encounters.

The significant relationship between ACEs and adolescent mental disorders and depression highlights the importance of early intervention strategies, especially initiatives at the family and community levels. Previous studies have confirmed the effectiveness of many family support programs in improving adolescent mental and behavioral health [34, 35]. Specifically, health education support is needed to raise parents' awareness of the negative consequences of ACEs, teach them how to communicate effectively with their adolescents, improve their parenting skills, and have them engage in stress management practices. Given that adolescents of racial/ethnic minorities and less-educated parents are more likely to experience family dysfunction, as noted in this study, these instructional efforts will be particular.

In this study, a unit change Oslo-3 Social Support Scale among adolescents decreases PHQ-9A score by −0.273 (*β* = −0.273, 95% CI −0.378, −0.168). In line with our studies, a study done in central Uganda and Malaysia [36, 37] shows that having low and moderate social support as measured by Oslo-3 is significantly associated with depression. Findings suggested that having good social support decreases the risk and severity of depression symptoms.

Depression prevalence found to be higher among grade 12 and 11 students. In contrary, the higher prevalence of depression among students of grades 11 and 12 may be due to stress related to performance in final board examinations and preparation for competitive examinations [38] .

## Figures and Tables

**Table 1:** Sociodemographic profile of adolescents who participated in the study.

Variables	Category	*N*	s(%)
Age	14	13	2.4
	15	84	15.4
	16	114	20.9
	17	153	28.0
	18	137	25.1
	19	45	8.2

Religion	Orthodox Christian	212	38.8
	Muslim	240	44.0
	Protestant	75	13.7
	Others	19	3.5

Occupation of father	Laborer	40	7.3
	Merchant	219	40.1
	Private	124	22.7
	Governmental	163	29.9

Educational status of father	Illiterate	40	7.3
	1-4	69	12.6
	5-8	124	22.7
	9-12	163	29.9

Substance use among adolescents	Cigarette smokers	5	0.9
	Alcohol drinkers	90	16.5
	Khat chewers	44	8.1
	Other illegal drugs	5	.9

Sex	Female	329	60.3
	Male	217	39.7

Grade	9	174	31.9
	10	153	28.0
	11	103	18.9
	12	116	21.2

School	Governmental	442	81.0
	Private	104	19.0

Occupation of mother	Laborer	34	6.2
	Merchant	93	17.0
	Private	137	25.1
	Government	147	26.9
	House wife	135	24.7

Educational status of mother	Illiterate	51	9.3
	1-4	96	17.6
	5-8	159	29.1
	9-12	138	25.3
	Certificate and above	102	18.7

Adolescents residence	Urban	450	82.4
	Rural	96	17.6

Other illegal drugs include ganja, shisha, and other substances or medication use.

**Table 2:** Cross-tabulation of ACE distribution by sex of respondents.

ACE characteristics		Boys (*N* = 214), %	Girls (*N* = 332), %	Overall (*N* = 546), %	*p* value
Emotional abuse		42.3	57.7	20.3	0.256
Physical abuse		40.7	59.3	15.8	0.422
Physical neglect		39.2	60.8	9.0	0.095
Emotional neglect		38.6	61.4	12.8	0.510
Sexual abuse		24.2	75.8	6.0	0.049
Household members substance abuse		51.9	48.1	9.5	0.035
Household members incarceration		35.5	64.5	13.9	0.283
Household members mental illness		40.7	59.3	4.9	0.508
Family violence		39.2	60.8	5.7	0.443
Parental divorce		31.4	68.6	15.8	0.068
For each ACE type category	Abuse	47.9	52.1	17.6	0.036
	Neglect	41.6	58.4	16.3	0.349
	Household dysfunction	45.0	55.0	18.3	0.115

**Table 3:** Effect of ACEs count on depression symptoms with cross-tabulation.

ACEs		Depression symptoms category						*p* value
	Category	Nondepressed (*N* = 393)		Depressed (*N* = 153)		Overall (*N* = 546)		
		Count	%	Count	%	Count	%	
ACE scores category	ACE = 0	216	80.3	53	19.7	269	49.3	<0.001
	ACE = 1 − 3	157	70.1	67	29.9	224	41.0	
	ACE > 3	20	37.7	33	62.3	53	9.3	

Type of adverse childhood experience	Abuse	64	66.7	32	33.3	96	17.6	<0.001
	Neglect	43	72.0	46	28.0	89	16.3	
	Household dysfunction	73	73.0	27	27.0	100	18.3	

**Table 4:** Final regression model for adolescents.

Model	Unstandardized *β*	Sig.	95.0% confidence interval for *β*	
			Lower bound	Upper bound
Constant	7.853	0.000	6.902	8.804
Childhood adverse experience	1.123	0.000	0.872	1.373
Excellent self-rated health	−2.621	0.000	−3.606	−1.637
Good self-rated health	−1.870	0.000	−2.794	−0.945
Grade 9 students	−1.878	0.000	−2.797	−0.960
Male gender	−1.107	0.007	−1.906	−0.308
Grade 10 students	−1.294	0.008	−2.251	−0.337
Rural residence	1.174	0.027	0.137	2.211
Oslo-3 Social Support	−0.273	0.032	−0.378	−0.168

## Data Availability

The datasets used and analyzed during the current study is available from the corresponding author and sent as supporting file.
